# An Experimental Enquiry Concerning the Presence of Alcohol in the Ventricles of the Brain, after Poisoning with That Liquid; Together with Experiments, Illustrative of the Action of Alcohol

**Published:** 1839-10

**Authors:** 


Art. XII.—*An Experimental Inquiry concerning the presence of Alcohol in the Ventricles of the Brain, after Poisoning with that liquid; together with Experiments, illustrative of the Physiological Action of Alcohol.* By John Percy, m.d.—*London*, 1839. 8vo, pp. 112.

We have already had occasion to notice, and with much commendation, more than one thesis, to which a gold medal was awarded by the medical faculty of the University of Edinburgh. The thesis before us is also a prize essay, by a graduate of the same University. Its chief value consists in the experiments, by which the question respecting the presence of alcohol in the brain, after poisoning with that fluid, is determined.

The general opinion has been—although the evidence on which such opinion rests is not extant—that in such cases alcohol might be found in the brain and ventricles after death. Dr. Percy has shown, beyond a doubt, that alcohol is taken up into the system; he having obtained it, by a process to be described, from the brains of dogs poisoned by it. But none of his experiments have enabled him to determine whether the fluid of the ventricles, under such circumstances, contains any alcohol. It would rather appear that there was some peculiar affinity between the substance of the brain and the spirit, especially as, after analyzing a much larger quantity of blood than can possibly exist in the cranium, he could generally obtain much more alcohol from the brain than from this quantity of blood. In his experiments, Dr. Percy also detected alcohol in the blood, urine, bile, and liver; and he connects the last fact with the frequency of hepatic disease in drunkards.

The following is the principle of the method adopted for obtaining the spirit from any fluid or organ to be examined. For a minute description we must refer to the work itself. The spirit is separated from the solid or fluid supposed to contain it, by distillation with water. The spirit is separated from the fluid, which distils over, by means of subcarbonate of potass; and the mode of testing this spirit (always small in quantity) is by its inflammability and its power of dissolving camphor. There is no doubt of the correctness of Dr. Percy's tests, nor any appearance of fallacy in his experiments or the conclusions which he draws from them. The essay contains the minute account of many experiments, the subjects being mostly dogs; and the spirit being injected into the stomach, jugular veins, or carotid arteries. The conclusions to which Dr. Percy's experiments have led him, respecting the modus operandi of alcohol are not of an exclusive character. He alludes to the discrepant opinions of the various writers on the action of poisons; and, in reply to that which maintains that the action of poisons depends exclusively on an impression upon the extremities of the nerves, he says

"We should certainly expect that this cerebral derangement would, in every instance, almost instantaneously succeed the exhibition of alcohol, especially when in large quantity, and in a concentrated form. On a careful review, however, of the experiments detailed, it will be observed, that generally an interval of several minutes elapsed before the slightest manifestation of cerebral derangement was afforded. Hence it is inferred that in some, or rather in the greater number of cases, absorption is required for the development of the narcotic effects of alcohol. . . . . In some of the experiments, on the other hand, total loss of sensibility and voluntary fever so instantaneously followed the introduction of the poison into the stomach, that we cannot conceive that absorption to a sufficient extent could possibly have been instantaneously effected. Hence there can be little doubt that alcohol can produce its fatal effect without being absorbed." (pp. 108-9.)

In these cases we must suppose the action to be upon the extremities of nerves. The following remarks are worthy of attention, as applicable to the modus operandi of alcohol.

"It has been objected, that the circumstance of intoxication being in some instances almost instantly abated by vomiting, is incompatible with the idea that alcohol may act by absorption directly upon the 'central organ of the nervous system.' But, independently of the fact that alcohol may be detected in the brain and blood, I may answer, *first*, that in dogs I have never (although I have watched with great attention) seen such immediate abatement, even after repeated and violent vomiting, as is generally represented to take place in the human subject; *secondly*, that, even admitting the correctness of the observation upon which the objection is founded, the very act of vomiting may probably be a sufficient stimulus to account for the subsequent relief; and this is supported by the current opinion, that vomiting is much more efficacious than complete evacuation by the stomach-pump; *thirdly*, that I recently witnessed a case of profound intoxication in a man, in which complete evacuation by the stomach-pump was not attended by any immediate abatement of the symptoms; indeed, the intoxication suffered no diminution for two or three hours afterwards, during which time the breath continued strongly alcoholic." (pp. 111-12.)

The experiments in Dr. Percy's essay detail with minuteness the effects produced upon animals by the presence of alcohol in their system, from the time of its introduction until death; and the appearances after death are likewise described. An investigation, conducted with similar care to that which we have just described, in which various individual substances should be made the object of experiment seems to be an object worthy of consideration, as likely to lead us somewhat farther onwards in the obscure path of the action of medicines. It is something for which we have to thank Dr. Percy, to have ascertained beyond a doubt the presence of alcohol in the brain; it is something more to have had it shown as a matter of high probability, that this spirituous fluid has apparently a considerable affinity for the cerebral substance. We know that medicinal substances have been detected in the blood, and that the action of many is more directed upon particular organs than others. How far this fact may be explained, on the supposition of a particular affinity of the medicine for the organ, is a subject well worthy of enquiry, and one which we should recommend Dr. Percy, with his aptitude for experimental investigation, carefully to perform.

